# Ethical Issues Linked to the Development of Telerehabilitation: a Qualitative Study

**DOI:** 10.5195/ijt.2021.6367

**Published:** 2021-06-22

**Authors:** Marin Guy, Alexandra Blary, Joel Ladner, Maxime Gilliaux

**Affiliations:** 1 Centre Aquitain Du Dos, Hôpital Privé Saint-Martin, Pessac, France; 2 La Musse Hospital and Physiotherapy School (Fondation La Renaissance Sanitaire), Clinical Research Department, Saint-SéBastien-De-Morsent, France; 3 Rouen University Hospital, Epidemiology and Health Promotion Department, Rouen, France; 4 Cetaps Laboratory, EA 3832, Rouen University, France

**Keywords:** Ethics, Physical Therapy Modalities, Telemedicine, Telerehabilitation

## Abstract

While telerehabilitation (TR) makes it possible to respond to many significant health system problems, TR still gives rise to debates, particularly concerning ethical issues. This qualitative study collected the opinions of stakeholders with varied profiles. A guided interview focused on discerning strategies that might foster the ethical deployment of TR. Such strategies were found to be linked to the decision-making of the public authorities, the role of scientific and professional bodies, the training of health professionals, and the management of patient information. Ethical issues relating to the development of TR included universal accessibility, patients’ free choice, respect of privacy, and professional confidentiality. The ethical development of TR can be fostered by the provision of information to stakeholders as well as reminding practitioners of the ethical framework that regulates medical practice.

Technologies applied to the world of health can be referred to as “e-health” (Haute Autorité de Santé, 2021). The development of e-health shows promise to address European public health challenges such as the aging of the population, the increase and burdens of chronic pathologies, and the uneven distributions of health care professionals (Sénat, 2019).

Nanotechnologies, biotechnologies, informatics and cognitive sciences (NBIC) are each showing the capacity to revolutionize our daily lives. The European Commission has therefore invited all member states to not only recognize the transformative potential of these technologies, but also to acknowledge the risks of such advances (Nordmann, 2004). It is undeniable that as e-health technologies develop, the management of rehabilitation patients will evolve.

Telerehabilitation (TR) employs information and communication technologies to conduct rehabilitation actions at a distance (Kaur et al., 2004). These include evaluations, tests, interventions, and follow-up activities. TR also makes it possible to offer consultations between professionals via remote expertise, and even to exchange clinical information about a patient between healthcare teams (Agence d'évaluation des technologies et des modes d'intervention en santé, 2006). The advantages of TR are closely akin to those of e-health and include the ability to provide access to healthcare in under-resourced areas.

TR can be used by many health professionals, including physicians in physical medicine and rehabilitation and general medicine, physiotherapists (i.e., physical therapists), occupational therapists, audiologists, speech-language pathologists, and neuropsychologists (Schmeler et al., 2009). TR is used by physiotherapists for various types of treatment such as neurological damages (e.g., stroke, head trauma), chronic diseases (e.g., chronic obstructive pulmonary disease, diabetes), and also in postoperative rehabilitation (e.g., post orthopedic surgery). Whether used in cases of trauma, cardiac, or respiratory rehabilitation, studies have shown the equal effectiveness of TR compared to care in in-person consultation (Chan et al., 2016); (Nelson et al., 2020; (Thomas et al., 2019).

TR can therefore be considered “relatively” equivalent to traditional rehabilitation care, taking into account the technical constraints imposed by geographic distance (Agence d'évaluation des technologies et des modes d'intervention en santé, 2006); (Association Médicale Mondiale, 2017). It must not mean a break or distancing from the patient (Conseil National de l'Ordre des Médecins, 2018). There is a risk of a breakdown in the equality of care (Conseil National de l'Ordre des Médecins, 2019) with discrimination between populations who may have access to in-person care as opposed to those who are cared for remotely. Thus, beyond the biomedical aspect, TR potentially disrupts the pathway to care and raises many ethical questions.

The World Medical Association defines ethics as “the study of morality, careful and systematic reflection and analysis of moral decisions and behaviors, past, present or future” (Association Médicale Mondiale, 2015). The World Health Organization (WHO) provides a framework for assessing any ethical problems that might arise and defines an appropriate course of action (Organisation mondiale de la Santé, 2009). Ethics is a complex construct since it applies where the law allows freedom of decision. The ethical principles recommended by WHO are: individual autonomy, beneficence, nonmaleficence and the equitable distribution of benefits and constraints (Organisation mondiale de la Santé, 2009).

A prior international literature review enabled us to demonstrate that recent studies on TR are mainly concerned with the medico-economic impact of TR (Haute Autorité de Santé, 2013) and that ethical issues to our knowledge are not or little addressed (Commission Européenne, 2010). The identification of these ethical issues is key to stimulating the optimal ethical development of TR.

The objective of this study was to identify the ethical issues linked to the development of TR and was based on a qualitative design. The study design was based upon “phenomenology,” to discern the experience of users and experts concerning a professional practice. The study sought meaning and understanding and involved the exploration of a topic or issue in depth (Letts et al., 2007).

## PARTICIPANTS

The inclusion criteria for participants were expertise in rehabilitation, TR, ethics, and the use of digital technology in healthcare. Five participants considered as “expert” were included: three experts who have published about TR (scientific, legal, deontological and ethical aspects), as well as two experts using TR (a rehabilitation practitioner and a patient). The characteristics of the recruited subjects are presented in Table 1.

**Table 1:** Characteristics of Included Experts

Expert	Gender	Profile and profession
Expert	Gender	Profile and profession
E1	M	Physiotherapist practicing TR
E2	F	Physiotherapist, President of the National Council of the Order of Physiotherapists (France)
E3	M	General practitioner, former president of the French Telemedicine Society and author of the book “Telemedicine, issues and practices” (Simon, 2015)
E4	M	Lecturer, philosopher and ethicist, lecturer at the Center for Medical Ethics and co-creator of the chair “Digital health: Ethics, law and governance”
E5	M	Patient using a telerehabilitation solution

*Note.* Abbreviations: E: Expert. M: Male. F: Female. TR: telerehabilitation

The five interviewees were drawn from a convenience sample. They had knowledge of the problem we wanted to explore, and some of them had acknowledged expertise. They were contacted by means of a standard contact email together with the request for consent of recording rights as well as the preamble text addressing the various concepts essential to the interview (definitions of remote rehabilitation and ethics). The recruitment and interview period extended from January 22, 2020 to March 25, 2020.

## DATA COLLECTION

A semi-structured interview guide was produced. Questions collected the knowledge, personal opinions and ideas of those interviewed on these topics. The interview guide consisted of a preliminary question (“Can you tell me about your professional background and your link with the topic we want to address today: telerehabilitation?”). Then, four questions on the main topics, and a concluding question (“To conclude, do you have further comments, or do you want to add something we haven't addressed?”). Stimulus questions were also prepared in advance.

To meet the main objective of this study (understanding the ethical issues of TR), the questions were:

- “How is this new practice, telerehabilitation, perceived from your point of view and that of your profession?”
- “In your opinion, what are the issues underlying the deployment of telerehabilitation?”
- “According to you, what are the ethical principles involved in the development and implementation of a TR program, both for the patient and the physiotherapist?”

To identify the means to be implemented to witness an ethical deployment of TR, the following question was asked: “How do you think we could achieve an ethical deployment of telerehabilitation?”

Questions were adapted according to the profile and the skills of the interviewees in order to optimize comprehension. Firstly, the word “telerehabilitation” could be replaced by “telemedicine”. Secondly, a specific version was created for the interview with the patient. The meaning of the questions had remained the same; we only changed a few words since the patient was not intervening in the context of a particular training/profession but for his personal experience.

The interviews were conducted by a health professional trained in conducting semi-structured interviews. The written and informed consent to participate in this study was obtained from all interviewees. As this study of human and social sciences aimed to evaluate the modalities of practice of health professionals, it did not fall under the provisions applicable to research on the human person. As a result, it was not the subject of a request for an opinion from an ethics committee.

Nevertheless, this study respected the regulations concerning the respect of personal data, in particular by drafting a procedure in accordance with the national Commission Nationale de l’Informatique et des Libertés” and European General Data Protection Regulation (GDPR) recommendations.

## DATA ANALYSIS

A telephone meeting was set up with each of the participants. All the interviews were recorded using a Smartphone and a Dictaphone. The recording of each interview was fully transcribed into a computer file using a word processor, in chronological order. The lines of these files were numbered. Each speech was then analyzed in three stages:

– a pre-analysis of the discourse consisting of a floating reading of what was said, making it possible to appropriate the discourse, to have an overall view of what was said and to grasp the general trend of the interviewee's opinion,
– a categorization of the discourse and a thematic indexing based on the identification of the elements of the conversation with regard to the themes targeted in the interview guide, and on the indexing of the latter in tables. This phase is a content analysis of the discourse. Elements of the discourse are then detached from the globality of the context in order to extract the themes, they are decontextualized. The passages of the argument that allow the research question to be answered are then retained and grouped in categorization tables by unit of meaning. Reliability is attested by the sentence snippets extracted from the discourse and juxtaposed with the identified themes,
– the inference and interpretation of the categorization tables allowed us to analyze the different statements collected in the tables, and to raise the important elements of the answers on the themes targeted by the questions.

Finally, it was realized the interpretation of the categorization tables. A directed content analysis approach was used to analyze the data and identify the themes. The data obtained from these various interviews were then analyzed; the common points tackled by several speakers were grouped together and compared with each other. The most recurrent ideas were developed in verbatim form.

## RESULTS

Five persons were included; their socio-demographic characteristics are presented in Table 1. The interviews lasted on average (± Standard deviation) 37 minutes (± 12 minutes). The professionals who responded to the interview promote and use telerehabilitation and defend it especially with the French public authorities. Persons (Expert (E) 1 and E2, Table 1) highlighted the differing opinions towards TR. Indeed, this tool turns out to be a practice that divides rehabilitators in France.

## ETHICAL ISSUES

The first ethical issue highlighted by four of the speakers (E1, E2, E3, E4) was the risk of reinforcing inequalities on the socio-economic level: although TR allows patients to benefit from rehabilitation care when a follow up in the consulting room and/or at home is complicated. The General Practitioner (GP) emphasized the risks of inequalities concerning access to innovation in the different French territories: *“[…] Mind that these innovations, I would say, benefit all regions"*.

E2 referred to access to digital coverage allowing access to technology; access to equipment is expensive: *“[…] You need equipment, you need investment, you need money. So, it should not be the door open to […] a two-speed rehabilitation.”*

In addition, E2 and E3 specified that TR may require the use of a third party to help the patient in this activity: “*[…] the need for equipment that we can use”* E2.

The second issue to be put forward concerned legislation and deontology: “*[…] When you are at a distance, you actually have to structure the legal framework well” E3.* Indeed, the philosopher (E4) underlined that TR is creating *“questions about collaboration and responsibilities, about the distribution of responsibilities which is completely new”*.

E4 believed that they need to be respected and even adapted to this new care environment that TR is shaping. By the way, the representative of the Order of Physiotherapist (E2) mentioned that *“[…] Our code of deontology is being uh adapted […] and we have already prepared texts, I believe from memory, for this, for these new issues”*

Issues concerning respect for privacy, data protection, autonomy and patient consent were mentioned by all participants: “*[…] I think you also have to be sensible because it shouldn’t be too intrusive either”* E2.

TR opens the door to the patient's home and personal space (E4). There is, according to the latter, a change of the living space into a care space and in the temporality of the care: *“[…] Digital technology really transforms the context of care, both spatially and temporally”* E4.

The last issue to be highlighted dealt with professional practices and the caregiver-patient relationship which have both changed, and particularly the risk of losing “human contact". This aspect is mentioned by the patient (E5): “*[…] For sure, well, you don't have human contact. That's just about the only thing missing“*.

TR and more generally new health technologies promote communication between the different healthcare professionals around the patient and multi-professional, inter-professional and intra-professional work (E1, E3): “*[…] Ability to communicate er between […] the different professions, around the patient and with the patient”* E1.

In addition, these technologies aim to make the patient an active partner in his health, which corresponds to “self-management” (*“[…] There are a lot of digital devices like this that tend to make the patient more active.”* E2) with medicine geared towards preventing and anticipating the onset of diseases according to the GP we interviewed. Nevertheless, the philosopher (E4) somehow feared a medicine that might increase the patient's vigilance, to the point of making him await a forthcoming disease: *“[…] preventive medicine, which takes the form of a medicine which, which, which turns the patient towards his future until sometimes making uh, projecting the healthy person towards a state of possible future disease”*.

### MEANS FOR THE ETHICAL DEVELOPMENT OF TELEREHABILITATION

Faced with these challenges, all participants agree that there is no single solution. Several issues are mentioned by the speakers to improve an ethical deployment of TR.

Some expect a lot from public authorities who are familiar with technical issues (digital and material coverage, etc.), particularly in relation to the challenges of health inequalities (E1, E2, E3).

Legislators could clearly define the distribution of professional responsibilities which are therefore modified by this tool (E3, E1): *“[…] we have to find a framework for it, er precise, er rational […] clearly show the points, the precise points to use this tool”* E1.

The representative of the Order of Physiotherapists (E2) suggests the possibility of creating new rights for patients, such as the right to disconnect: *“[…] Perhaps we should also have the right to disconnect”)*.

The GP (E3) mentions the role of scientific bodies who must inform their members, in particular with the publication of guides for professional practice: *“[…] There has to be a scientific authority who can uh enlighten the members of their corporation, let's say on the successes and failures of such and such developments in professional practices”*.

The role of the Order of Physiotherapists has also been regularly mentioned, (E3, E2, E4), because it is up to this body to guide the profession in carrying out acts of TR and to enforce current deontological rules (*“[…] And of course then there are the Councils of the respective Orders of, of all the professionals submitted to the Orders who… Who in any case ensure the respect of ethics”* E2).

Finally, it seems essential to train health professionals in the use of these tools, but more generally in the transformation of the health system which is currently in progress (E1, E2, E4): “*[…] it requires training, training in the use of the tool, training in communication, uh, that refers to lifelong training”* E1. These health professionals (E1, E3) also have a duty to train.

It is important to inform patients of the advantages and limitations of this tool. Health professionals also have a role to play in providing feedback to legislators. For their part, it is essential that the patient be informed of the possibilities available to him (E3, E4): *“[…] on the patient side, they must be properly informed, they must give their consent to a new treatment solution”* E3.

*Two experts emphasized that RT should be considered a complementary practice: “[…] Tele-rehabilitation must not be exclusive”* E1; *“[…] It will not be a total substitution of current care but will be complementary practices”* E3.

## DISCUSSION

Many institutions, research committees and scientists are currently turning to the use of telemedicine and telerehabilitation in the treatment of certain pathologies. National and international plans are in place to deploy the use of telemedicine in certain situations (Commission Européenne, 2010). Some authorities are considering the publication of guidelines for the use of this tool (Agence d'évaluation des technologies et des modes d'intervention en santé, 2006); (Brennan et al., 2010). Nevertheless, in these “guides” to develop telerehabilitation projects, the ethical aspects are briefly mentioned and often without justification. Thus, in order to guide telerehabilitation projects on these aspects, it seemed necessary to find out how telerehabilitation brought certain ethical aspects into play, and what means were envisaged in order to overcome these problems.

The objectives of this study were to identify the ethical issues linked to the development of TR and the means to be implemented to consider its ethical deployment. These results demonstrated that they are not so easy to answer, and no solutions are raised, when ethical issues are discussed. In the end, it is not a decision that must be taken in the face of the issues raised, but rather an ethical reasoning that would accompany the development of TR.

Organizational, economic, political and legal constraints have made it possible to highlight the ethical issues that they underlie. Indeed, these constraints can be a source of inequality in access to care, but also in the free choice of the patient, with respect for private life and professional secrecy. The European Union, in its final report of the “Methotelemed” project concerning Model for Assessment of Telemedicine (MAST), conducted a review of the literature on the various aspects taken into account and evaluation criteria for a telemedicine project (Commission Européenne, 2010). This report underlines the issue of equity among different societal groups for the access to this technology, it is essential that this tool be universally accessible. They also evoke the transfer of responsibility with the active participation of the patient in self-care. The patient is placed at the heart of this process and it is up to the patient to give consent to the use of this type of tool. Responsibility for data protection remains with healthcare and legal professionals in choosing the tool to be used.

In this “Methotelemed” report, the understanding by the patient and the patient's relatives of the application of telemedicine is also mentioned. The World Medical Association (WMA), in its ethical position for telemedicine, even adds that it is essential to ensure that users, whether they are patients or healthcare professionals, know how to use this type of digital tool. The WMA also emphasizes that telemedicine should not be chosen solely for economic reasons (reduction in the costs of treatment). This study showed that TR does not require new ethical and deontological principles to be defined. Indeed, the WMA, in its ethical position concerning telemedicine, specifies that the major ethical principles specific to medical practice also apply to the practice of telemedicine. On the other hand, it is advisable to ensure that the pre-existing principles of rehabilitation are respected in these new situations of remote care. This aspect seems to be in line with the recommendations issued by the European Council of Medical Orders (CEOM). Indeed, this council affirmed, during its declaration in Bari in June 2014 that “the ethical and deontological principles in force remain and apply to this practice of medicine” (Conseil Européen Ordres des Médecins, 2014). The application of these principles in this new context of care is essential to the ethical use of TR. This application may also give rise to new laws, as was previously suggested with the right for the patient to disconnect (Ministère des Solidarités et de la Santé, 2018) or as was the case for the GDPR (Code de la santé publique, 2019).

Deployment of TR that would be respectful of ethical principles therefore requires an ethical reflection carried out by the various actors so that they are aware of the issues and the points of vigilance underlying the program. It would therefore be a question of informing all the actors involved in a TR program. The ethical framework could thus be applied at three levels. First, even more attention must be paid to the quality of communication. This assumes that health professionals follow training sessions that will have made them aware of communication tools, the motivational link and the impact of turning to such a practice. Thus, training physiotherapists in using digital health is essential (Brennan et al., 2010). The National Council of General Practitioner, in the aforementioned white paper on connected health, suggests that “training should not focus on the tool but on its ethical and deontological integration into medical practice itself, for the benefit of the patient” (Conseil National de l’Ordre des Médecins, 2015). Second, ethical stake could be also exercised at the level of industrial health promoters seeking to develop an application of TR. It could be suggested that any developed application be evaluated by an ethics committee. Thus, using an interview with the representatives of the project, for example, the committee speakers would seek to highlight the ethical principles involved in this process. This committee could be made up of different representatives of the competent bodies (scientific, deontological and professional). The ethical, deontological and legal frameworks could be explained and a debate could be organized in order to collect the opinions of the various actors and it could lead to a validation of the project by the ethics committee (Code de la santé publique, 2019). This would then make it possible to promote the setting up of TR in clinical routine. Third, ethical support for these projects could also be considered. This would make it possible, with the help of patients, to set the limits of such a practice. It could also be considered to create an informative booklet available to stakeholders in a TR program in order to raise ethical questions among its readers about the measures they want to put in place. But also, an “ethical” certification label of TR programs could be suggested, as proposed by Lasbordes (2009) in his report on telehealth. Different application labels integrate this ethical consideration in their evaluation process (Observatoire de la santé visuelle et auditive, 2016).

## CLINICAL AND RESEARCH PERSPECTIVES

These results could be applied in clinics and used for future research. At clinical level, the results of this study encourage healthcare professionals to train in the use of these new technologies and to find out about the context in which they are developed, in order to use telerehabilitation in the best possible conditions to ensure quality and secure patient care. At research level, the position of healthcare professionals vis-à-vis this technology, their desire to use this tool in their future practice, their opinion about this tool could be interesting aspects to study through a quantitative epidemiological study. The quantitative approach would allow large samples to be obtained and the results to be statistically processed. Another quantitative epidemiological study could be undertaken on a population of patients in order also to discover their points of view in relation to this technology, but also their perception of the rehabilitation profession and what they expect from it. It might even be relevant to correlate their perception of this profession with their point of view vis-à-vis TR. Moreover, it would also be appropriate to question the influence that the deployment of this technique can have on the general model of rehabilitation service delivery as well as on the perception of the latter.

In addition, it would be wise to focus on the feedback from patients and rehabilitators following the COVID-19 pandemic. This evaluation could be based on numerous criteria such as efficiency, socio-economic interest, relationship, acceptability, the methods used, the perception according to these methods and the evolution of the vision of the profession of re-educator. Finally, it could be useful to produce an evaluation grid of ethical criteria to be respected so that a TR project can obtain an ethical “certification label” as mentioned above, with transversal support from expert consultants who could come together under the same format as the already existing ethics committees.

## LIMITATIONS

This study was carried out using a qualitative methodology, with the advantages and limitations that this generates. The choice of the target population was guided since the participants intervened within the framework of their knowledge and experiences with respect to TR, which may have led to a selection bias. This selection made it possible to have informed opinions as well as expertise on a subject that is under development. The results are not representative of the profession or of the general population but allow us to investigate a topical and innovative subject from a reflective aspect. Even if semi-structured interviews seemed to be the most suitable procedure to answer our research question, a future quantitative study could be carried out, in particular with healthcare professionals and patients, in order to complete the results from the interviews with the therapist and the patient (see Perspectives).

It is possible that the small sample of professionals we included deprived us of some concepts and made it more difficult to extrapolate the results, and also meant an under-representation of other professionals who could have been included as well.

On the collegial aspect of ethical decision-making, it could have been interesting to organize in parallel with these individual interviews, a focus group where all these stakeholders would have met and would have been able to discuss the questions asked. Nevertheless, the choice was made to collect these different opinions individually, then to rely on the author of this work to correlate these discourses. The idea was that speakers should speak freely and independently of the judgment or influence of a group hearing. A survey of several people from the same profession or sharing the same position with respect to TR would have yielded weighted results.

Also, one of the questions in the interview guide describes telerehabilitation as a “new practice.” It would be more appropriate to use the term “service delivery model” to be consistent with existing bodies of research and prevailing terminology.

## CONCLUSION

This study highlights the ethical issues linked to the development of TR, such as the risk of reinforcing socio-economic inequalities or the impact on professional practices and the caregiver-patient relationship. If we are to witness an ethical deployment of TR, the involvement of public health authorities as well as the emphasis on the role of scientific bodies, the training of health professionals, and information from the patient need to be implemented.

In addition, the deployment of TR, which would be respectful of ethical principles, requires reflection to be carried out by the various actors so that they are aware of the issues and points of vigilance underlying the program in question. This support could be done by experts, in a labeling procedure.

Future studies, in particular quantitative ones, could be carried out in order to assess the position of healthcare professionals and patients with respect to TR, their desire to use this tool in their future practice or their opinion concerning this service delivery model.
